# Health’s influence on alcohol use—a longitudinal study of working adults in Sweden

**DOI:** 10.1093/eurpub/ckag037

**Published:** 2026-03-22

**Authors:** Erica Jonsson, Devy L Elling, Jonas Landberg, Magnus Helgesson, Andreas Lundin, Emelie Thern

**Affiliations:** Institute of Environmental Medicine, Karolinska Institutet, Stockholm, Sweden; Institute of Environmental Medicine, Karolinska Institutet, Stockholm, Sweden; Department of Public Health Sciences, Stockholm University, Stockholm, Sweden; Department of Public Health and Caring Science, Uppsala University, Uppsala, Sweden; Department of Clinical Neuroscience, Karolinska Institutet, Stockholm, Sweden; Center for Epidemiology and Society Medicine, Region Stockholm, Stockholm, Sweden; Institution for Global Public Health, Karolinska Institutet, Stockholm, Sweden; Epidemiology of Psychiatric Conditions, Substance Use and Social Environment, Karolinska Institutet, Stockholm, Sweden; Institute of Environmental Medicine, Karolinska Institutet, Stockholm, Sweden

## Abstract

While alcohol’s health effects are well documented, less is known about how health influences alcohol use and whether this varies by socioeconomic position (SEP). This study investigated the association between health-related quality of life (HRQoL), mental health, and alcohol use, and whether SEP moderates these associations. Baseline data from 7097 participants in the 2010 Stockholm Public Health Cohort were used. The exposures were HRQoL and mental health (good, moderate, poor); Outcomes (2014) were heavy episodic drinking (HED: ≥5 units/≥2 times/month) and heavy drinking (men: ≥21 units/week; women: ≥14 units/week). Logistic regression estimated odds ratios (OR), with interaction assessed using relative excess risk of interaction (RERI) and attributable proportion (AP). Joint exposure analyses used good health and high SEP as the reference group. Compared with good HRQoL, moderate (OR: 1.26, 95% CI: 1.02–1.56) and poor HRQoL (OR: 1.39, 95% CI: 1.08–1.78) were associated with higher odds of heavy drinking. Moderate HRQoL and low SEP had increased odds of HED (OR: 1.48, 95% CI: 1.02–2.15) and heavy drinking (OR: 1.62, 95% CI: 1.01–2.60), with evidence of additive interaction (RERI: 0.79; AP: 0.49). Mental health findings were less consistent: good mental health and low SEP was associated with increased HED (OR: 1.35), while moderate mental health and intermediate SEP was associated with decreased HED (OR: 0.66). Findings suggest a dose-response relationship between HRQoL and self-reported heavy drinking and an interaction between moderate HRQoL and low SEP. Associations with mental health were weaker and inconsistent.

Key points

- This study suggested a potential dose-response relationship between health-related quality of life and self-reported heavy drinking
- Evidence for socioeconomic position as a moderator was limited, except between moderate health-related quality of life and low socioeconomic position
- Workers’ health-related quality of life and socioeconomic context should be considered when forming alcohol prevention strategies

## Introduction

The global burden of disease estimates show that alcohol is one of the most important risk factors for loss of healthy life years [1]. Extensive research has examined the adverse effects of alcohol use on health, finding health-related costs like alcohol-related diseases, injuries, and addiction accounting for a significant share of alcohol’s 100 billion SEK economic burden in Sweden [2]. However, the reverse relationship—how health factors influence alcohol consumption—has received comparatively less attention.

Alcohol use is an important cause of social inequality in health and can reinforce the negative effects of poor health [3]. If poor health also increases alcohol use, they may amplify each other and lead to work-related consequences such as absenteeism, sickness absence, and early labour market exit [2]. The risk factors of alcohol use and health factors may have differential vulnerabilities across socioeconomic position (SEP) [4]. While individuals with high SEP may consume similar or greater amounts of alcohol, those with lower SEP experience more severe alcohol-related consequences [3], in part due to a higher incidence of heavy episodic drinking (HED) [3, 5]. Low SEP is also associated with greater alcohol-attributable mortality in adults [6], suggesting that the same level of consumption may result in disproportionate harm depending on socioeconomic context. Examining whether SEP and health jointly affect alcohol use may also lead to a greater understanding of who is more vulnerable in the working population.

### Health-related factors, socioeconomic position, and alcohol use

The relationship between alcohol consumption and mental health appears complex and bidirectional and may vary across socioeconomic groups. A meta-analysis found depression to be related to future alcohol use as a form of self-medication [7], while another study found no evidence of self-medication and that depression decreased the level of alcohol consumption [8]. An investigation of the temporal relationship between mental health and alcohol use found that mental health influences alcohol use more than the reverse [9], with good mental health predicting reductions in drinking and poor mental health maintaining heavy use [9]. Supporting this perspective, a systematic review utilizing Mendelian randomization found causal evidence that mental disorders lead to increased alcohol use, while no evidence was identified for the reverse direction [10]. Importantly, these associations may vary across SEP. For example, lower SEP is associated with higher rates of mental health issues in youth [11], and some evidence suggests that poor mental health is associated with hazardous drinking in both low and high SEP groups, but not in intermediate SEP [12]. These findings highlight the need to consider SEP when evaluating mental health’s role in alcohol use.

Although mental health appears to be an important determinant of alcohol use, it represents only one dimension of overall well-being. HRQoL entails the physical, social and mental well-being of a person [13]. There is a certain overlap between mental health and HRQoL regarding depression and anxiety, yet they capture different aspects of health [14]. Evaluating both measures of health can help disentangle which aspects of health affect drinking more.

Several studies have found an association between increased alcohol use and reduced HRQoL [15–18]. However, as many of these studies were cross-sectional, they offer limited insight into the direction of the relationships [16, 18]. There is some evidence to indicate that HRQoL influences alcohol use. For instance, poor HRQoL during adolescence has been shown to predict an earlier onset of alcohol use and the development of alcohol use disorder (AUD) [19]. Whereas, improving HRQoL appeared to reduce alcohol use and support AUD recovery [20]. Notably, HRQoL demonstrates a clear socioeconomic gradient [21], and socioeconomic factors may improve HRQoL in individuals with an AUD [20]. Moreover, Ummels et al. (2022) identified a bidirectional association between anxiety disorder (anxiety is a dimension used to measure HRQoL) and AUD, and anxiety disorder’s risk on AUD was sensitive to sociodemographic factors [22]. Although SEP may moderate the relationship between HRQoL and alcohol use, existing studies often focused more on HRQoL outcomes rather than drinking behaviour and typically examined AUDs rather than self-reported alcohol use.

Investigating whether health factors such as HRQoL and mental health are associated with alcohol use may help inform the development of targeted interventions, especially if certain socioeconomic groups within the working population are more vulnerable than others. To the best of our knowledge, previous studies have rarely focused on working populations, where the interplay between health, alcohol use, and SEP may have distinct implications. This study, therefore, aims to evaluate HRQoL and mental health’s association with alcohol use and examine whether SEP moderates the association in the working population.

## Methods

### Study design and population

This study used data from the Stockholm Public Health Cohort (SPHC), which sends a survey out every 4 years to a random sample in Stockholm County and conducts follow-ups for each cohort in successive data collections [23]. The sample is meant to be representative of the adult population in the Stockholm area and is linked to several national registers [23]. This study used the SPHC cohort with a baseline in 2010 and follow-up in 2014 (*n* = 18 517; response rate 60%). The study population’s inclusion criteria were individuals at baseline living in Sweden, aged 25–60 years old, employed (until further notice, time-limited or self-employed), and had complete variable data. Minimum age was determined as individual SEP is typically less established before the age of 25. To address reverse causality, we excluded individuals who, prior to baseline, had an alcohol diagnosis based on international classification of disease (ICD-10) in the national patient register [mental and behavioural disorders due to alcohol (F10), alcoholic liver disease (K70), toxic effects of alcohol (T51), and other alcohol-related diagnoses: E24.2, G31.2, G62.1, G72.1, I42.6, K29.2, K85.2, K86.0, O35.4, R78.0, Z04.0, Z71.4, and Z72.1] [24]. The national patient register includes Inpatient and Specialised Outpatient Care Registers [25]. After applying the inclusion criteria, the study base was 7097 participants (see Figure S1 in Supplementary for details).

The data was linked to the national register The Longitudinal Integrated Database for Health Insurance and Labor Market Studies (LISA) to obtain information on the highest attained education level and income. Data was linked and pseudonymized by Statistics Sweden. The Swedish Ethical Review Authority approved ethical approval with reference number 2023-00493-01.

### Exposure

The exposures were self-reported HRQoL and mental health. HRQoL was measured using the EuroQol group EQ-5D-3L instrument, consisting of five dimensions regarding mobility, self-care, usual activity, pain/discomfort, and anxiety/depression [26]. Each dimension was scored between 1–3 points, where a score of 1 indicated ‘no problems’, 2 indicated ‘some problems’ and 3 indicated ‘severe problems’. The total score of HRQoL was calculated (5–15 points) and were categorized based on the following cutoffs [27]: good HRQoL (5 points), moderate HRQoL (6 points), and poor HRQoL (≥7 points). Individuals defined as having poor HRQoL had an average score of 7.34.

Mental health was measured using the General Health Questionnaire (GHQ-12) [26], which comprised of 12 items on mental well-being. In the survey, positive and negative outlooks on mental well-being were measured using six items on a four-point scale, respectively. Based on the presentation of the response alternatives, each alternative was scored according to the binary method (0–0–1–1), where 0 point indicated no distress and 1 point indicated distress among the participants [28]. A total score for mental health (0–12 points) was calculated and categorized into three categories: good mental health (≤2 points), moderate mental health (3–5 points), and poor mental health (≥6 points) [29]. Individuals defined as having poor mental health had an average score of 7.50. We adapted the cut-offs for the exposure variables to be staggered in order to capture varying levels of severity and assess potential dose-response relationships.

### Outcomes

Alcohol use, measured in 2014, was assessed through two different self-reported drinking behaviours, heavy drinking and HED. The drinking behaviours were examined separately as they differ in their sociodemographic patterns [5]. HED was defined as drinking more than 5 standard units of alcohol on one occasion at least 2 times per month [30]. Heavy drinking was defined as drinking 14 standard units or more per week for females and 21 standard units or more for males [30].

### Moderator

SEP was derived from self-reported occupation, which was categorized by Statistics Sweden into six groups according to the Swedish socioeconomic classification of occupation [31]. In this study, SEP was further categorized into three groups to increase power: low SEP (unskilled worker, skilled worker), intermediate SEP (low and middle non‐manual workers, self‐employed) and high SEP (high non‐manual workers). Self-employed individuals were categorized as having intermediate SEP because it was not possible to determine whether they engage in manual or non-manual work.

### Covariates

Covariates were chosen based on previous literature on health and hazardous drinking [9, 17, 20, 32]. Sociodemographic characteristics, including sex (male, female), age (25–30, 31–40, 41–50, 51–60 years), SEP (low, intermediate, high) and migration background (Swedish-born, foreign-born) were collected from the SPHC survey. Information on the highest level of education (primary ≤9 years, secondary 10–12 years, university >12 years), and annual income (in SEK: <250 000, 250 000–349 999, 350 000–449 999, 450 000–549 999, ≥550 000) were obtained from the LISA register. Moreover, information regarding available personal support during crisis (yes/no), former HED (yes/no) and former heavy drinking (yes/no) was derived from the survey. In cases where information on highest education attainment was missing from the LISA register (*n* = 11), this was complemented by self-reported education from the SPHC survey.

### Statistical analysis

The study population’s characteristics were presented with frequency distributions across the different SEP groups. Logistic regression was used to measure the association between exposures, SEP and outcomes, with results presented as odds ratios (OR) and 95% confidence intervals (CI). First, a crude model was fitted. Model 1 adjusted for sociodemographic factors and personal support during a crisis. The fully adjusted model (model 2) was further adjusted for prior drinking behaviour to evaluate the impact of prior drinking versus the other covariates.

Joint exposure variables were created to assess additive interaction between health exposures (HRQoL, mental health) and SEP on alcohol outcomes (HED, heavy drinking). Additive interaction was prioritized over multiplicative due to greater public health relevance [33]. A joint exposure variable also enables a common reference group (good health, high SEP) [34]. Moderation by SEP was evaluated with the relative excess risk due to interaction (RERI) using Rothman’s formula (RERI = OR11 − OR10 − OR01 + 1) for individuals with low SEP and moderate or poor health. The attributable proportion due to the interaction (AP = RERI/OR11) was calculated for each RERI as well as 95% CI using the DELTA method [33].

Several sensitivity analyses were performed. First, individuals with heavy drinking or HED at baseline were excluded, as alcohol use may affect health [32]. Second, those who reported a chronic illness—defined as a long-lasting disease, mishap, handicap, or other long-lasting health problem affecting workability—were removed, given its strong association with both alcohol use and health [35]. Third, individuals who were unemployed or on sickness absence in the follow-up were excluded, as these are known risk factors for hazardous alcohol use [36, 37].

The analysis was conducted using Stata Statistical Software, release 17.

## Results

Table 1 summarizes the study population characteristics of 7097 working individuals aged 25–60 in the 2010–2014 SPHC, presented for the total population and by SEP. A majority of the study population were female (55%), over 40 years old (68%), Swedish-born (89%), and reported they had personal support during a crisis (91%). Over half of the study population reported intermediate SEP (56%), followed by high SEP (28%), and the fewest reported low SEP (15%). A socioeconomic gradient appeared with low SEP more common among low education, low income, prior drinking, HED, heavy drinking, and low HRQoL, while mental health showed no clear SEP differences.

**Table 1.** Descriptive characteristics of the study population, stratified by socioeconomic position (SEP)

	Total *n* (%)	Low SEP *n* (%)	Intermediate SEP *n* (%)	High SEP *n* (%)
Total	7097 (100)	1063 (15)	4018 (57)	2016 (28)
Sex				
Male	3177 (45)	542 (51)	1602 (39)	1033 (51)
Age				
25–30	558 (8)	105 (9)	300 (7)	153 (7)
31–40	1745 (25)	225 (21)	961 (23)	559 (27)
41–50	2462 (35)	318 (29)	1454 (36)	690 (34)
51–60	2332 (33)	415 (39)	1303 (32)	614 (30)
Migration background				
Swedish-born	6295 (89)	851 (80)	3600 (89)	1844 (91)
Highest attained education				
Primary	378 (5)	151 (14)	205 (5)	22 (1)
Secondary	2531 (36)	744 (70)	1503 (37)	284 (14)
University	4188 (59)	168 (16)	2310 (57)	1710 (84)
Income				
0–249 999	1133 (16)	313 (29)	720 (17)	100 (5)
250 000–349 999	2017 (28)	514 (48)	1240 (30)	263 (13)
350 000–449 999	1735 (24)	180 (17)	1094 (27)	461 (22)
450 000–549 999	991 (14)	42 (4)	536 (13)	413 (20)
550 000+	1221 (17)	14 (1)	428 (10)	779 (38)
Personal support	6464 (91)	941 (88)	3672 (91)	1851 (91)
Former HED	779 (11)	359 (33)	885 (22)	367 (18)
Former heavy drinker	1611 (23)	165 (15)	434 (10)	180 (8)
HED	1401 (20)	325 (30)	749 (18)	327 (16)
Heavy drinker	674 (10)	145 (13)	368 (9)	161 (8)
HRQoL				
Good	3701 (52)	434 (40)	2077 (51)	1190 (59)
Moderate	2189 (31)	378 (35)	1201 (29)	610 (30)
Poor	1207 (17)	251 (23)	740 (18)	216 (10)
Mental health				
Good	5897 (84)	902 (84)	3324 (82)	1671 (82)
Moderate	671 (9)	81 (7)	387 (9)	203 (10)
Poor	529 (7)	80 (7)	307 (7)	142 (7)

HED: heavy episodic drinking; HRQoL: health-related quality of life.

### Main effects

The individual associations of HRQoL, mental health, and SEP with outcomes HED and heavy drinking are presented in Table 2. Poor HRQoL was associated with 20% increased odds of HED, although this association attenuated after adjusting for all covariates. In relation to heavy drinking, both moderate and poor HRQoL were associated with higher odds in the fully adjusted model, indicating a dose-response pattern (moderate OR: 1.26, 95% CI: 1.02–1.56; poor OR: 1.39, 95% CI: 1.08–1.78).

**Table 2.** Odds ratios (OR) and 95% confidence intervals (CI) of heavy episodic drinking (HED) and heavy drinking given health-related quality of life (HRQoL), mental health, and socioeconomic position (SEP).

		HED (OR 95% CI)		
	*n* (%)	Crude	Model 1	Model 2
HRQoL				
Good	666 (18)	1.00	1.00	1.00
Moderate	458 (21)	1.21 (1.06–1.38)	1.23 (1.07–1.41)	1.09 (0.92–1.29)
Poor	277 (23)	1.36 (1.16–1.59)	1.37 (1.16–1.63)	1.20 (0.97–1.48)
Mental health				
Good	1192 (20)	1.00	1.00	1.00
Moderate	108 (16)	0.76 (0.61–0.94)	0.87 (0.70–1.09)	0.81 (0.62–1.06)
Poor	101 (19)	0.93 (0.74–1.17)	1.11 (0.88–1.41)	0.97 (0.72–1.29)
SEP				
High	327 (16)	1.00	1.00	1.00
Intermediate	749 (19)	1.18 (1.03–1.36)	1.05 (0.89–1.23)	0.97 (0.80–1.18)
Low	325 (31)	2.27 (1.91–2.71)	1.65 (1.32–2.05)	1.27 (0.97–1.66)

		**Heavy drinking (OR 95% CI)**		
		
		**Crude**	**Model 1**	**Model 2**

HRQoL				
Good	283 (8)	1.00	1.00	1.00
Moderate	237 (11)	1.52 (1.27–1.82)	1.40 (1.16–1.68)	1.26 (1.02–1.56)
Poor	154 (13)	1.87 (1.52–2.31)	1.62 (1.30–2.01)	1.39 (1.08–1.78)
Mental health				
Good	556 (9)	1.00	1.00	1.00
Moderate	58 (9)	0.95 (0.71–1.26)	1.03 (0.77–1.37)	0.99 (0.71–1.37)
Poor	60 (11)	1.31 (0.98–1.74)	1.38 (1.03–1.85)	1.19 (0.85–1.67)
SEP				
High	161 (8)	1.00	1.00	1.00
Intermediate	368 (9)	1.16 (0.96–1.41)	1.01 (0.82–1.26)	0.96 (0.75–1.24)
Low	145 (14)	1.82 (1.43–2.31)	1.25 (0.93–1.68)	1.09 (0.75–1.24)

Model 1: adjusted for age, sex, income, migration background, education, SEP, support; Model 2: adjusted for age, sex, income, migration background, education, SEP, support, prior HED, prior heavy drinking.

No clear association was observed between mental health and HED or heavy drinking in the fully adjusted model. Compared to good mental health, moderate mental health indicated a negative association with HED (model 2 OR: 0.81, 95% CI: 0.62–1.06). For heavy drinking, poor mental health initially indicated increased odds, yet this association attenuated in the fully adjusted model.

The associations of SEP with HED and heavy drinking were presented to distinguish individual associations from joint exposure associations. Compared to high SEP, low SEP displayed an increased odds of HED, which attenuated in the fully adjusted model (OR: 1.27 95% CI: 0.97–1.66). Low SEP only showed increased odds for heavy drinking in the crude model.

### Joint exposure of HRQoL and SEP

Table 3 displays the joint associations of HRQoL and SEP with HED and heavy drinking. Compared to individuals with good HRQoL and high SEP, those with moderate HRQoL and low SEP had increased odds of HED in the fully adjusted model (OR: 1.48, 95% CI: 1.02–2.15). This group also showed evidence of additive interaction, with a RERI of 0.58 (95% CI: 0.02–1.13) and an AP of 0.39 (95% CI: 0.07–0.71). Associations for other HRQoL and SEP combinations attenuated after full adjustment.

**Table 3.** Odds ratios (OR) and 95% confidence intervals (CI) of health-related quality of life (HRQoL) and socioeconomic position (SEP) on heavy episodic drinking (HED) and heavy drinking.

			HED (OR 95% CI)		
		*n* (%)	Crude	Model 1	Model 2
HRQoL/SEP					
Good	High	187 (16)	1.00	1.00	1.00
	Intermediate	356 (17)	1.11 (0.91–1.35)	1.01 (0.82–1.25)	0.84 (0.65–1.07)
	Low	123 (28)	2.12 (1.63–2.75)	1.40 (1.04–1.90)	1.07 (0.74–1.54)
Moderate	High	104 (17)	1.10 (0.85–1.43)	1.15 (0.88–1.51)	0.83 (0.60–1.14)
	Intermediate	229 (19)	1.26 (1.02–1.56)	1.24 (0.98–1.57)	0.96 (0.73–1.27)
	Low	125 (33)	2.65 (2.03–3.45)	1.83 (1.35–2.49)	1.48 (1.02–2.15)
Poor	High	36 (17)	1.07 (0.73–1.59)	1.25 (0.84–1.88)	1.01 (0.62–1.63)
	Intermediate	164 (22)	1.53 (1.21–1.93)	1.53 (1.18–1.99)	1.14 (0.84–1.56)
	Low	77 (31)	2.37 (1.74–3.24)	1.63 (1.14–2.32)	1.14 (0.74–1.75)

			**Heavy drinking (OR 95% CI)**		
			
			**Crude**	**Model 1**	**Model 2**

HRQoL/SEP					
Good	High	79 (7)	1.00	1.00	1.00
	Intermediate	163 (8)	1.20 (0.91–1.58)	1.10 (0.82–1.49)	1.01 (0.72–1.41)
	Low	41 (10)	1.47 (0.99–2.18)	1.08 (0.70–1.67)	0.75 (0.46–1.23)
Moderate	High	56 (9)	1.42 (0.99–2.03)	1.42 (0.99–2.03)	1.08 (0.72–1.63)
	Intermediate	121 (10)	1.58 (1.17–2.12)	1.41 (1.03–1.95)	1.15 (0.80–1.66)
	Low	60 (16)	2.65 (1.85–3.80)	1.92 (1.28–2.88)	1.62 (1.01–2.60)
Poor	High	26 (12)	1.92 (1.20–3.07)	2.01 (1.25–3.24)	1.74 (1.01–3.02)
	Intermediate	84 (11)	1.80 (1.31–2.48)	1.56 (1.10–2.23)	1.15 (0.77–1.72)
	Low	44 (18)	2.99 (2.01–4.44)	2.12 (1.36–3.31)	1.61 (0.96–2.70)

Measure of HRQoL and SEP interaction on additive scale (after full adjustments):

HED moderate HRQoL/low SEP RERI (95% CI) = 0.58 (0.02 to 1.13), AP (95% CI) = 0.39 (0.07 to 0.71). HED poor HRQoL/low SEP RERI (95% CI) = 0.07 (−0.62 to 0.75), AP (95% CI) = 0.06 (−0.53 to 0.65). Heavy drinking moderate HRQoL/low SEP RERI (95% CI) = 0.79 (0.07 to 1.51), AP (95% CI) = 0.49 (0.13 to 0.84). Heavy drinking poor HRQoL/low SEP RERI (95% CI) = 0.11 (−1.01 to 1.23), AP (95% CI) = 0.07 (−0.62 to 0.76). Model 1: adjusted for age, sex, income, migration background, education, SEP, support; Model 2: adjusted for age, sex, income, migration background, education, SEP, support, prior HED, prior heavy drinking.

For heavy drinking, individuals with poor HRQoL and high SEP had increased odds compared to the reference group (OR: 1.74, 95% CI: 1.01–3.02). In other SEP groups with poor HRQoL, the association attenuated in model 2. Individuals with moderate HRQoL and low SEP had increased odds of heavy drinking (OR: 1.62, 95% CI: 1.01–2.60), with evidence of additive interaction (RERI: 0.79, 95% CI: 0.07–1.51; AP: 0.49, 95% CI: 0.13–0.84).

### Joint exposure of mental health and SEP

Table 4 presents the joint associations of mental health and SEP with HED and heavy drinking. Compared to individuals with good mental health and high SEP, those with good mental health and low SEP had increased odds of HED (OR: 1.35, 95% CI: 1.02–1.79). In contrast, moderate mental health in the intermediate SEP group was associated with decreased odds of HED (OR: 0.66, 95% CI: 0.44–0.98).

**Table 4.** Odds ratios (OR) and 95% confidence intervals (CI) of mental health and socioeconomic position (SEP) on heavy episodic drinking (HED) and heavy drinking

			HED (OR 95% CI)		
		*n* (%)	Crude	Model 1	Model 2
Mental health/SEP					
Good	High	277 (17)	1.00	1.00	1.00
	Intermediate	632 (19)	1.18 (1.01–1.38)	1.05 (0.88–1.24)	0.96 (0.78–1.18)
	Low	283 (31)	2.30 (1.90–2.78)	1.66 (1.32–2.10)	1.35 (1.02–1.79)
Moderate	High	29 (14)	0.84 (0.55–1.26)	0.86 (0.57–1.30)	0.81 (0.50–1.32)
	Intermediate	58 (15)	0.89 (0.55–1.27)	0.81 (0.59–1.11)	0.66 (0.44–0.98)
	Low	21 (26)	1.76 (1.05–2.94)	1.28 (0.75–2.18)	1.24(0.64–2.42)
Low	High	21 (15)	0.87 (0.54–1.41)	0.87 (0.54–1.42)	0.78 (0.43–1.40)
	Intermediate	59 (19)	1.20 (0.88–1.64)	1.07 (0.78–1.49)	0.88 (0.59–1.31)
	Low	21 (26)	1.79 (1.07–3.00)	1.31 (0.77–2.24)	1.14 (0.58–2.24)

			**Heavy drinking (OR 95% CI)**		
			
			**Crude**	**Model 1**	**Model 2**

Mental health/SEP					
Good	High	131 (8)	1.00	1.00	1.00
	Intermediate	300 (9)	1.17 (0.94–1.44)	1.03 (0.81–1.30)	0.99 (0.75–1.29)
	Low	125 (14)	1.89 (1.46–2.45)	1.30 (0.95–1.79)	1.13 (0.79–1.63)
Moderate	High	15 (7)	0.94 (0.54–1.63)	1.05 (0.60–1.84)	1.01 (0.54–1.87)
	Intermediate	36 (9)	1.21 (0.82–1.77)	1.17 (0.78–1.75)	1.06 (0.66–1.68)
	Low	7 (9)	1.11 (0.50–2.46)	0.85 (0.89–2.80)	0.77 (0.31–1.94)
Low	High	15 (11)	1.39 (0.79–2.44)	1.58 (0.89–2.80)	1.49 (0.77–2.88)
	Intermediate	32 (10)	1.37 (0.91–2.06)	1.33 (0.87–2.04)	1.05 (0.64–1.72)
	Low	13 (16)	2.28 (1.23–4.24)	1.81 (0.94–3.47)	1.42 (0.65–3.11)

Measure of mental health and SEP interaction on additive scale (after full adjustments):

HED moderate mental health/low SEP RERI (95% CI) = 0.08 (−0.84 to 0.99), AP (95% CI) = 0.06 (−0.64 to 0.77). HED poor mental health/low SEP RERI (95% CI) = 0.02 (−0.88 to 0.92), AP (95% CI) = 0.01 (−0.77 to 0.79). Heavy drinking moderate mental health/low SEP RERI (95% CI) = −0.36 (−1.34 to 0.61), AP (95% CI) = −0.47 (2.02 to 1.08). Heavy drinking poor mental health/low SEP RERI (95% CI) = −0.19 (−1.63 to 1.24), AP (95% CI) = −0.14 (−1.22 to 0.95). Model 1: adjusted for age, sex, income, migration background, education, SEP, support; Model 2: adjusted for age, sex, income, migration background, education, SEP, support, prior HED, prior heavy drinking.

There were no clear associations between mental health, SEP, and heavy drinking. Elevated estimates observed for individuals with low SEP attenuated after adjustment for covariates in models 1 and 2, and no statistically significant interactions were detected.

### Sensitivity analysis

The sensitivity analysis further confirmed the robustness of the main analysis. Some of the results attenuated yet still indicated similar estimates (Supplementary Tables 1–3).

## Discussion

This study examined how HRQoL and mental health were associated with HED and heavy drinking, and whether SEP moderated these relationships. The associations varied by both drinking behaviour and health factor. Poor HRQoL was strongly associated with heavy drinking, suggesting a dose-response relationship across levels of HRQoL severity. HRQoL also showed evidence of an interaction with SEP and both drinking behaviours. In contrast, the association between mental health and alcohol use was less consistent and appeared more strongly influenced by SEP, with no evidence of interaction between the two.

### Quality of life

In the total study population, HRQoL suggested a dose-response relationship with both outcomes, however, it was clearer for heavy drinking. While the prevalence of HED was higher across all exposure groups, heavy drinking demonstrated a stronger and more consistent association with declining HRQoL. These findings align with previous research showing reduced HRQoL is associated with heavy drinking, HED, and AUD [15, 16, 19].

Findings further suggest that SEP may modify the association between HRQoL and both drinking behaviours. Notably, among individuals with poor HRQoL, those with high SEP had elevated odds of heavy drinking- an association not observed in other SEP strata. This may be in part due to high SEP individuals generally drinking more frequently [3]. Even when their HRQoL is reduced, individuals with high SEP may be more likely to heavily drink compared to other SEP strata due to their greater resources, social opportunities and normalized use of alcohol in higher-income settings [4]. Alternatively, lower SEP’s elevated baseline levels of alcohol use, potentially diluted the relative impact of poor HRQoL. The attenuation observed after adjusting for prior drinking in model 2 may, to some extent, control for habitual drinking patterns.

The additive interaction between moderate HRQoL and low SEP was associated with both HED and heavy drinking, indicating that individuals with low SEP may be more vulnerable to alcohol-related risks even at moderate levels of HRQoL. One possible explanation is that HRQoL may be closely related to working conditions, particularly in low SEP occupations that are more physically demanding and associated with greater pain, discomfort, and alcohol use [21]. This socioeconomic gradient was reflected in the distribution of HRQoL across SEP groups, in contrast to the more uniform distribution observed for mental health.

The finding that increased alcohol use occurred among individuals with moderate, but not poor, HRQoL in the low SEP group warrants further investigation. It may suggest a non-linear relationship when SEP is factored in. This pattern may reflect health limitations restricting alcohol use among those with poor HRQoL, or that stress and coping behaviours manifest differently across health levels [38]. Moderate HRQoL may reflect a threshold where individuals remain functional but experience enough distress or discomfort to be vulnerable to maladaptive coping, such as alcohol use [39].

### Mental health

Poor mental health did not show a clear association with alcohol use in the total population. Increased odds of HED were only seen among individuals with low SEP. These findings contrast with prior studies that have reported a positive association between poor mental health and alcohol use. Several studies have found poor mental health to influence alcohol use [9, 10, 12]. However, methodological differences such as cross-sectional designs [12] and the use of clinical diagnoses rather than self-reported symptoms [10] may partly explain these discrepancies. The GHQ-12 instrument used in this study may capture milder forms of psychological distress, which may not reflect the severity of mental ill health typically linked to increased alcohol use [7]. Another explanation may be a selection bias from evaluating the working population; Individuals who have poorer health, and also drink more, may have been excluded as they could not work [32].

Among individuals with low SEP, those with good mental health were more likely to engage in HED, while the association weakened with poorer mental health. This may suggest that individuals with low SEP and good mental health are more susceptible to external social influences that promote drinking [3, 4].

### Strengths and limitations

A strength of this study was the use of a longitudinal design which enabled the evaluation of a temporal association with different measurements of health and alcohol. Categorizing the exposure variables into multiple groups, instead of a binary method was also a strength as it revealed dose-response relationships. This categorization, however, does limit comparability with other studies. Another factor that may limit comparability was the use of a higher cut-off score compared to previous research for heavy drinking and HED, yet this enabled us to better capture individuals with more hazardous alcohol use, where associations with health were more likely to be detectable.

The main limitation of this study was the inability to determine with certainty the direction of the association between health factors and alcohol use. A feedback loop between these variables has been noted in the literature [9, 22], which would be difficult to disentangle without repeated measurements. To address potential reverse causality, we conducted a sensitivity analysis excluding participants with HED or heavy drinking at baseline. Although this reduced power, many estimates remained similar, supporting the robustness of our findings. Additionally, the four-year follow-up may limit the certainty of temporal associations, as intervening factors, including health at follow-up, could influence the observed relationships.

The analysis was conducted on persons with complete data, which may have introduced selection bias. Individuals with poor health or high alcohol use may not have completed the whole survey or underreported their use, potentially attenuating the estimates [40]. Evaluating a working population may also have introduced a health selection bias and attenuated the association [37]. Nonetheless, given the study’s inclusion criteria, the findings should be generalizable to workers of a similar demographic in Sweden, and likely other high-income countries.

## Conclusion

This study indicates a dose-response relationship between HRQoL and self-reported heavy drinking in the working population. Although evidence for SEP as a moderator was limited, the observed interaction between moderate HRQoL and low SEP underscores the importance of addressing both health status and socioeconomic context in workplace alcohol prevention efforts. Integrating targeted screening and support into occupational health care check-ups may offer a practical avenue for early identification and intervention. In contrast, associations with mental health were less consistent, likely reflecting the use of a milder measure of psychological distress. Future research should further explore this relationship across varying levels of mental health severity.

## Supplementary Material

ckag037_Supplementary_Data

## Data Availability

The data used in this article are not publicly available due to sensitive information. Data was provided by the Centre for Epidemiology and Social Medicine, Region Stockholm.
