# Supplementary material for: Health’s influence on alcohol use—a longitudinal study of working adults in Sweden
Source: Eur J Public Health. 2026 Mar 22;36(2):ckag037. doi: 10.1093/eurpub/ckag037 (PMC13017360; doi:10.1093/eurpub/ckag037)
Supplement: ckag037_Supplementary_Data [file ckag037_supplementary_data.zip › ejph-2025-09-om-0786-File003.docx]

Supplementary tables

Table 1: Odds ratios (OR) and 95% confidence intervals (CI) of heavy episodic drinking (HED) and heavy drinking given health-related quality of life (HRQoL), mental health, and socioeconomic position (SEP) when individuals unemployed and on sickness absence at follow up were removed (n=6970)

|  | HED | Heavy  drinking |  | HED | Heavy drinking |
| --- | --- | --- | --- | --- | --- |
| Mental health |  |  | **HRQoL** |  |  |
| Good | 1.00 | 1.00 | Good | 1.00 | 1.00 |
| Moderate | 0.82 (0.63-1.08) | 1.00 (0.72-1.39) | Moderate | 1.11 (0.94-1.32) | 1.29 (1.04-1.60) |
| Poor | 0.98 (0.73-1.31) | 1.23 (0.87-1.74) | Poor | 1.18 (0.96-1.46) | 1.44 (1.11-1.86) |
| Mental health/SEP |  |  | **HRQoL/SEP** |  |  |
| Good/  high SEP | 1.00 | 1.00 | Good/high SEP | 1.00 | 1.00 |
| Good/  intermediate SEP | 0.98 (0.79-1.20) | 1.01 (0.77-1.33) | Good/  intermediate SEP | 0.83 (0.65-1.07) | 1.04 (0.74-1.46) |
| Good/ low SEP | 1.25 (0.94-1.68) | 1.09 (0.75-1.58) | Good/ low SEP | 1.06 (0.74-1.53) | 0.74 (0.44-1.23) |
| Moderate/  high SEP | 0.92 (0.56-1.50) | 1.04 (0.56-1.94) | Moderate/ high SEP | 0.82 (0.59-1.13) | 1.14 (0.76-1.73) |
| Moderate/  intermediate SEP | 0.73 (0.49-1.09) | 1.06 (0.66-1.71) | Moderate/  intermediate SEP | 0.99 (0.75-1.32) | 1.21 (0.84-1.75) |
| Moderate/low SEP | 1.18 (0.59-2.36) | 0.82 (0.32-2.07) | Moderate/ low SEP | 1.51 (1.03-2.20) | 1.61 (0.99-2.60) |
| Poor/high SEP | 0.85 (0.46-1.54) | 1.53 (0.79-2.98) | Poor/high SEP | 1.01 (0.62-1.65) | 1.81 (1.03-3.16) |
| Poor/  intermediate SEP | 1.02 (0.68-1.54) | 1.10 (0.67-1.82) | Poor/  intermediate SEP | 1.12 (0.82-1.54) | 1.23 (0.82-1.85) |
| Poor/low SEP | 1.24 (0.63-2.45) | 1.43 (0.64-3.19) | Poor/low SEP | 1.13 (0.73-1.74) | 1.62 (0.95-2.76) |

Adjusted for age, sex, income, migration background, education, SEP, support, prior HED, prior heavy drinking

Table 2: Odds ratios (OR) and 95% confidence intervals (CI) of heavy episodic drinking (HED) and heavy drinking given health-related quality of life (HRQoL), mental health, and socioeconomic position (SEP) when removing individuals with chronic illness (n=6190)

|  | HED | Heavy drinking |  | HED | Heavy drinking |
| --- | --- | --- | --- | --- | --- |
| Mental health |  |  | **HRQoL** |  |  |
| Good | 1.00 | 1.00 | Good | 1.00 | 1.00 |
| Moderate | 0.93 (0.69-1.24) | 1.04 (0.73-1.50) | Moderate | 1.04 (0.86-1.24) | 1.23 (0.99-1.54) |
| Poor | 1.11 (0.80-1.54) | 1.10 (0.73-1.64) | Poor | 1.27 (0.98-1.63) | 1.29 (0.95-1.76) |
| Mental health/SEP |  |  | **HRQoL/ SEP** |  |  |
| Good/high SEP | 1.00 | 1.00 | Good/high SEP | 1.00 | 1.00 |
| Good/ intermediate SEP | 0.95 (0.76-1.18) | 0.96 (0.72-1.28) | Good/ intermediate SEP | 0.86 (0.67-1.11) | 1.06 (0.75-1.49) |
| Good/ low SEP | 1.22 (0.89-1.66) | 1.09 (0.74-1.62) | Good/ low SEP | 1.08 (0.74-1.57) | 0.77 (0.45-1.29) |
| Moderate/ high SEP | 0.91 (0.53-1.56) | 1.08 (0.56-2.07) | Moderate/ high SEP | 0.83 (0.60-1.17) | 1.22 (0.80-1.86) |
| Moderate/ intermediate SEP | 0.74 (0.48-1.14) | 1.10 (0.67-1.82) | Moderate/ intermediate SEP | 0.94 (0.70-1.26) | 1.13 (0.77-1.67) |
| Moderate/ low SEP | 2.21 (1.06-4.60) | 0.61 (0.18-1.98) | Moderate/ low SEP | 1.38 (0.92-2.08) | 1.54 (0.92-2.56) |
| Poor/high SEP | 0.80 (0.42-1.53) | 1.22 (0.57-2.59) | Poor/ high SEP | 0.98 (0.55-1.76) | 1.68 (0.86-3.29) |
| Poor/ intermediate SEP | 1.21 (0.77-1.91) | 0.89 (0.49-1.61) | Poor/ intermediate SEP | 1.16 (0.80-1.67) | 0.96 (0.59-1.55) |
| Poor/low SEP | 1.44 (0.64-3.27) | 1.69 (0.67-4.28) | Poor/low SEP | 1.59 (0.94-2.68) | 2.06 (1.11-3.84) |

Adjusted for age, sex, income, migration background, education, SEP, support, prior HED, prior heavy drinking

Table 3: Odds ratios (OR) and 95% confidence intervals (CI) of heavy episodic drinking (HED) and heavy drinking given health-related quality of life (HRQoL), mental health, and socioeconomic position (SEP) when removing former drinkers (n=5266)

|  | HED | Heavy drinking |  | HED | Heavy drinking |
| --- | --- | --- | --- | --- | --- |
| Mental health |  |  | **HRQoL** |  |  |
| Good | 1.00 | 1.00 | High | 1.00 | 1.00 |
| Moderate | 0.70 (0.45-1.07) | 0.61 (0.30-1.21) | Moderate | 1.12 (0.87-1.44) | 1.28 (0.89-1.86) |
| Poor | 1.21 (0.81-1.80) | 1.17 (0.66-2.09) | Low | 1.34 (0.99-1.81) | 1.24 (0.78-1.96) |
| Mental health/SEP |  |  | **HRQoL/ SEP** |  |  |
| Good/ high SEP | 1.00 | 1.00 | Good/ high SEP | 1.00 | 1.00 |
| Good/ intermediate SEP | 0.94 (0.69-1.27) | 1.23 (0.77-1.97) | Good/ intermediate SEP | 0.81 (0.57-1.56) | 1.10 (0.62-1.93) |
| Good/ low SEP | 1.45 (0.96-.21) | 1.72 (0.89-3.31) | Good/low SEP | 1.06 (0.62-1.83) | 1.45 (0.62-3.40) |
| Moderate/ high SEP | 0.73 (0.33-1.62) | 0.27 (0.04-1.99) | Moderate/ high SEP | 0.72 (0.43-1.21) | 0.77 (0.34-1.76) |
| Moderate/ intermediate SEP | 0.56 (0.29-1.09) | 1.17 (0.52-2.67) | Moderate/ intermediate SEP | 0.93 (0.62-1.39) | 1.74 (0.96-3.13) |
| Moderate/ low SEP | 1.35 (0.54-3.40) | 1 | Moderate/ low SEP | 1.90 (1.14-3.18) | 1.61 (0.67-3.89) |
| Poor/ high SEP | 1.15 (0.54-2.47) | 1.84 (0.69-4.89) | Poor/ high SEP | 1.31 (0.70-2.45) | 1.87 (0.78-4.49) |
| Poor/ intermediate SEP | 1.23 (0.70-2.18) | 1.47 (0.64-3.38) | Poor/ intermediate SEP | 1.16 (0.73-1.83) | 1.21 (0.59-2.50) |
| Poor/ low SEP | 1.46 (0.58-3.70) | 0.65 (0.08-5.06) | Poor/low SEP | 1.46 (0.79-2.69) | 1.68 (0.64-4.41) |

Adjusted for age, sex, income, migration background, education, SEP, support, prior HED, prior heavy drinking
