# Supplementary figures and images for: Health’s influence on alcohol use—a longitudinal study of working adults in Sweden
Source: Eur J Public Health. 2026 Mar 22;36(2):ckag037. doi: 10.1093/eurpub/ckag037 (PMC13017360; doi:10.1093/eurpub/ckag037)

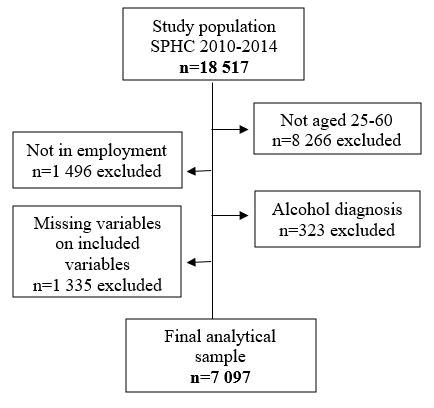

Supplement: ckag037_Supplementary_Data [file ckag037_supplementary_data.zip › ejph-2025-09-om-0786-File004.tiff]
